# Epizootic Spread of Schmallenberg Virus among Wild Cervids, Belgium, Fall 2011

**DOI:** 10.3201/eid1812.121067

**Published:** 2012-12

**Authors:** Annick Linden, Daniel Desmecht, Rosario Volpe, Marc Wirtgen, Fabien Gregoire, Jessica Pirson, Julien Paternostre, Deborah Kleijnen, Horst Schirrmeier, Martin Beer, Mutien-Marie Garigliany

**Affiliations:** Author affiliations: University of Liège, Liège, Belgium (A. Linden, D. Desmecht, R. Volpe, M. Wirtgen, F. Gregoire, J. Pirson, J. Paternostre, D. Kleijnen, M.-M. Garigliany);; Friedrich-Loeffler-Institut, Greifswald-Insel Riems, Germany (H. Schirrmeier, M. Beer)

**Keywords:** Schmallenberg, Bunyaviridae, epidemiology, wild cervids, red deer, roe deer, emerging, viruses, Belgium

## Abstract

Schmallenberg virus was detected in cattle and sheep in northwestern Europe in 2011. To determine whether wild ruminants are also susceptible, we measured antibody seroprevalence in cervids (roe deer and red deer) in Belgium in 2010 and 2011. Findings indicated rapid spread among these deer since virus emergence ≈250 km away.

During summer and fall of 2011, a nonspecific febrile syndrome among adult dairy cows in northwestern Europe was reported. During November 2011, an enzootic outbreak causing fetal death or neurologic signs in newborn lambs, kids, and calves emerged throughout several countries in Europe. Both syndromes were associated with the genome of a new Shamonda/Sathuperi-like orthobunyavirus named Schmallenberg virus (SBV) in the blood (adults) or central nervous system (newborns) (*1*,*2*). Susceptibility of wild ruminants can be expected on the basis of the behavior of related viruses of the Simbu serogroup. Therefore, we measured seroprevalence of antibodies against SBV in wild red deer (*Cervus elaphus*) and roe deer (*Capreolus capreolus*) and looked for the viral genome in fetuses from pregnant deer found dead.

## The Study

**Figure 1:**
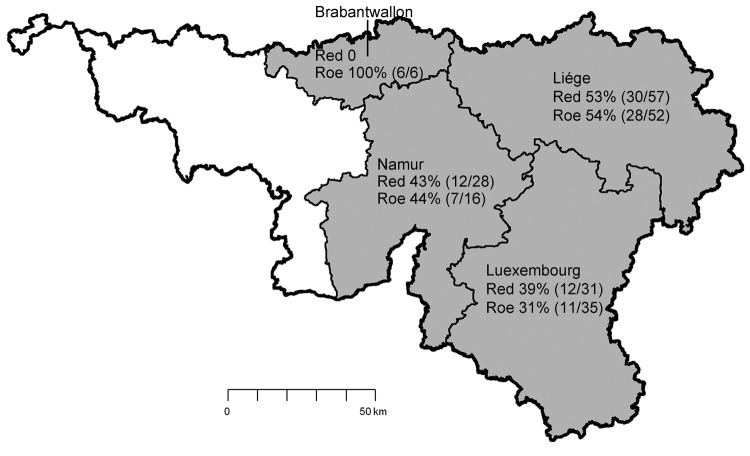
Location of 4 provinces in southeast Belgium (shaded) where 524 wild cervids (313 red deer and 211 roe deer) were killed during hunting seasons 2010 and 2011 and sampled. Seroprevelance for Schmallenberg virus is shown for each of the 225 deer killed in 2011. Source: Institut Géographique National, Brussels, Belgium, 2001.

Blood samples were collected during postmortem examination of 313 red deer and 211 roe deer shot during the 2010 and 2011 hunting seasons. The 524 samples were randomly collected during October–December from 35 hunting estates in 4 of the 5 provinces in southern Belgium (Figure 1). The animals’ sex; age; body condition; and macroscopic aspects of hooves, mucosae, and internal organs were recorded. IgG against the recombinant nucleoprotein of the emerging SBV was detected by using an ELISA kit (ID Screen Schmallenberg Virus Indirect, version 1; ID.vet Innovative Diagnostics, Montpellier, France). Results are expressed as percentages of the reference signal yielded by the positive control serum; serologic status is defined as negative (<60%), doubtful (60%–70%), or positive (>70%). Neutralizing antibodies against SBV were sought as described (*3*) in subsets of roe deer serum (IgG-negative and IgG-positive according to ELISA), and a linear relationship between percentages and reciprocal neutralizing titers was found. In addition, necropsies were performed on 22 fetuses and 5 newborn red deer fawns; brain samples were tested for SBV genomic RNA and cellular β-actin transcripts by reverse transcription quantitative PCR (*3*). Contingency tables were analyzed by using χ^2^ analysis to detect associations between seroconversion and species, sex, age, sampling location, and sampling date. Significance level was p<0.05.

No gross lesions compatible with any disease were found in any deer. All 299 serum samples collected during the fall of 2010 were negative for IgG against SBV. However, among the 225 samples from deer killed in 2011, seroprevalence was 43.1% (95% CI 36.6%–49.6%). No significant association was found between species and seroconversion: 40.5% (95% CI 31.6%–49.5%) among red deer and 45.9% (95% CI 36.5%–55.2%) among roe deer; p = 0.42. Acquired immunity against SBV was thus already high, suggesting that SBV had quickly spread since its emergence ≈250 km northeast during late summer 2011.

**Figure 2:**
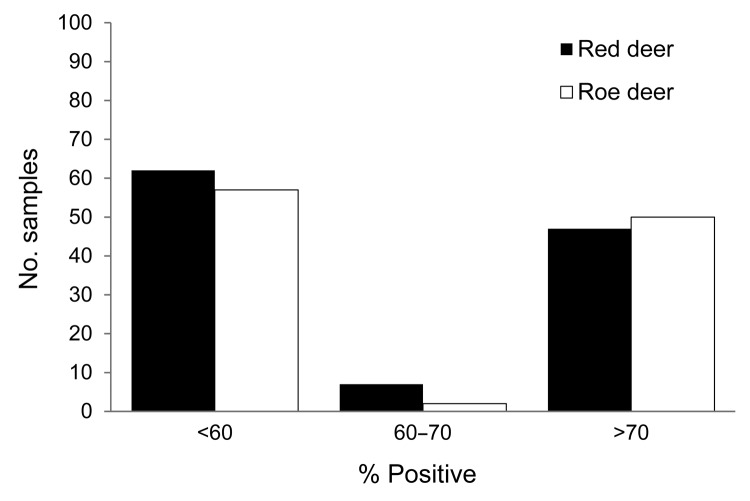
Frequency distribution of the results yielded by indirect ELISA for detecting IgG targeting recombinant nucleoprotein of emerging Schmallenberg virus in serum samples collected from 116 red deer and 109 roe deer in southeast Belgium during the fall of 2011. Results are expressed as percentages of the reference signal yielded by the positive control serum. Serologic status is defined as negative (<60%), doubtful (60%–70%), or positive (>70%).

A significant association between month of sampling and seroconversion was detected for both deer species (p = 0.0016 and 0.0083 for red and roe deer, respectively). Seroprevalence increased during weeks 40–50 of 2011: for red deer, 20.0% (95% CI 8.3%–31.7%) in October, 52.6% (95% CI 36.8%–68.5%) in November, and 54.6% (95% CI 37.6%–71.5%) in December and for roe deer, 34.0% (95% CI 20.5%–47.6%) in October, 49.1% (95% CI 35.6%–62.5%) in November, and 88.9% (95% CI 68.4%–100%) in December, thus suggesting that the virus had circulated in the areas sampled until at least mid-November (Figure 2).

This late circulation of virus might be surprising because biting midges of the genus *Culicoides*, which reportedly transmit SBV (*4*), are not usually active during cold months. However, during fall 2011, temperatures in the region were substantially higher than normal (*5*) and thus compatible with persistent wild-ruminant exposure to biting midges until mid-December. No association was found between seroconversion and sex of the deer (p = 0.71 and 0.85 for red and roe deer, respectively), age (p = 0.99 and 0.24), and location of sampling (p = 0.47 and 0.23). These results suggest a similar level of exposure to infected vectors and a similar degree of susceptibility to infection among all animals in the study area (13,058 km^2^).

In most animals that had been found dead, gross lesions were consistent with trauma (e.g., fractures, hematomas, hemoperitoneum/thorax, ruptured spleen) suggestive of impact against a vehicle. No fetus or newborn showed morphologic alterations of the neck, trunk, or limbs suggestive of arthrogryposis. No macroscopic abnormalities were seen in the cerebral cortex, cerebellum, and spinal cord. All β-actin–positive samples of these 27 fetuses and newborns remained negative for SBV RNA. Unfortunately, postmortem decay rendered fetal serum not suitable for analysis.

## Conclusions

SBV infects wild cervid populations, and infected insect vectors were homogeneously distributed over southern Belgium in the fall of 2011. Emergence probably took place in 2011. However, because seroprevalence was already 20% in red deer and 34% in roe deer during October and because our results show that the proportion of the infected population increased exponentially during October–December, we suggest that the virus began circulating months earlier than the currently believed August/September (*3*). We recently showed that among the fetuses of pregnant cows that were infected after the establishment of the first placentome, 28% were infected and that an arthrogryposis/hydranencephaly syndrome follows if transplacental virus transmission occurs before fetuses are immunocompetent (*6*). For this study, no feedback from forest rangers, no macroscopic observations, and no PCR results suggested transplacental contamination. However, aborted fetuses and stillborn and distorted nonviable newborn fawns are almost impossible to collect in the wild (quickly eaten by scavengers), and the absence of SBV-specific genetic material or morphologic alterations at necropsy are not evidence of noninfection. Therefore, no objective facts confirm or refute transplacental transfer.

Because the virus can infect the fetus only after the first placentome has developed and because roe deer embryos remain in diapause until January (*7*), it is unlikely that SBV has contaminated many roe deer fetuses. Because 90% of roe deer were already SBV positive in mid-December and because circulating antibodies prevent transplacental passage of the closest phylogenetic relatives of the virus (*8*), we suggest that roe deer fetuses were probably not infected. On the contrary, red deer mate in September, and the first functional placentome is established by the end of October (*9*); thus, 80% of pregnant red deer were exposed to the emerging virus when placental transfer was possible. Furthermore, 35% of pregnant red deer were infected in November and December, i.e., after establishment of the first placentome and before the fetus was immunocompetent. By extrapolating the rate of transplacental infection among cattle (*6*), we determined that 28% of these pregnancies resulted in contamination of the fetus, i.e., 10%, of expected pregnancies. Because unrestricted replication of Simbu-like viruses occurs in the central nervous system of immunologically incompetent ruminant fetuses (*1*), which can lead to a typical arthrogryposis/hydranencephaly syndrome, a 10% loss among fawns can be expected in 2012.

In the same geographic area, 5 years apart, 2 arboviruses have emerged: bluetongue virus serotype 8 (BTV-8) during the summer of 2006 and SBV during the summer of 2011. For each virus, *Culicoides* spp. midges function as vectors and infect sheep, goats, cattle, and red deer. Although most (>50%) red deer seroconverted against BTV-8, only a few (<3%) roe deer sampled in the same places and at the same time were BTV-8-positive (*10*), which sharply contrasts with the SBV seroconversion rates reported here. This finding invalidates the assumption that less exposure of roe deer to infected midge bites explains the almost complete absence of seroconversion against BTV-8 in this species. The emergence of SBV thus reveals the existence of roe deer–specific anti–BTV-8 host factors, posing a fascinating question.
